# Seroprevalence of infectious pathogens of zoonotic and veterinary importance in wild ruminants from Slovenia

**DOI:** 10.3389/fvets.2024.1415304

**Published:** 2024-06-10

**Authors:** Diana Žele Vengušt, Brane Krt, Rok Blagus, Gorazd Vengušt, Petra Bandelj

**Affiliations:** ^1^Institute of Pathology, Wild Animals, Fish and Bees, Veterinary Faculty, University of Ljubljana, Ljubljana, Slovenia; ^2^Institute of Microbiology and Parasitology, Veterinary Faculty, University of Ljubljana, Ljubljana, Slovenia; ^3^Institute for Biostatistics and Medical Informatics, University of Ljubljana, Ljubljana, Slovenia; ^4^Faculty of Mathematics, Natural Sciences and Information Technologies, University of Primorska, Koper, Slovenia

**Keywords:** wild ruminants, roe deer, red deer, serology, infectious diseases, *Toxoplasma gondii*, risk factors, One Health

## Abstract

**Introduction:**

Wildlife represents an increasingly important source of pathogens of medical and veterinary importance. Surveillance in wildlife offers an insight on current epidemiological status of selected pathogens and help to prevent spillovers to humans and livestock.

**Material and methods:**

Our study included 312 wild ruminants belonging to five species: Roe deer (*n* = 134), red deer (*n* = 113), Alpine chamois (*n* = 53), European mouflon (*n* = 10) and Alpine ibex (*n* = 2). Seven pathogens that may have profound effect on human/livestock health and economic viability of the farms were tested using serological methods.

**Results:**

Antibodies against *Toxoplasma gondii, Neospora caninum, Coxiella burnetii, Brucella* spp., *Chlamydophila abortus, Mycobacterium avium* subsp. *paratuberculosis* (MAP) and *Mycobacterium bovis* were detected in 34.62% (108/312), 0.96% (3/312), 2.24% (7/312), 0, 0.96% (3/312), 0, 0.64% (2/312) of animals tested, respectively. Because of low prevalences, risk factors were assessed only for *T. gondii*. Sex (female>male) and species (roe deer>red deer, roe deer>Alpine chamois) were significantly associated with the *T. gondii* positive outcome, while age was not.

**Discussion:**

Adult males had the lowest *T. gondii* prevalence which offers future research opportunities. The lower seroprevalence of most investigated pathogens suggests game meat, if properly cooked, as being relatively safe for human consumption. This is the first study investigating the seroprevalence and associated risk factors of selected pathogens in wild ruminants in Slovenia.

## 1 Introduction

Wild animals act as reservoirs for numerous diseases and can play different roles in the epidemiology of various pathogens. The recent outbreaks of infectious diseases in wildlife worldwide emphasize the importance of ensuring constant surveillance not only of animal pathogens but also of potential zoonotic agents (1). Diseases deriving from wildlife were recognized as a major cause of mortality and loss of biodiversity in animals, but they also have a devastating economic impact (2). Moreover, most emerging infectious diseases in humans are zoonoses or of animal origin (3). The significant impact of infectious diseases in wildlife has also been recognized by the World Organization for Animal Health (WOAH) and the World Health Organization (WHO), which have increased their efforts to establish early detection mechanisms for important zoonotic and conservation pathogens (4). In Slovenia, as in the whole of Europe, a significant increase in the population and geographical distribution of all wild ungulate species has been observed after the Second World War (5, 6). The roe deer (*Capreolus capreolus*) is the most widespread wild ungulate species in Slovenia, followed by other wild ruminants such as the red deer (*Cervus elaphus*), the Alpine chamois (*Rupicapra rupicapra*), the European mouflon (*Ovis aries musimon*) and the Alpine ibex (*Capra ibex*) (4). A seropositive outcome to zoonotic pathogens is common among wild ruminants (7–14). The most common pathogens, such as the unicellular parasites *Toxoplasma gondii* and *Neospora caninum*, pose a considerable risk as they increase the abortion rate in wild and domestic ruminants. In addition, *T. gondii* is recognized as a major public health concern (15–19). The prevalence of *T. gondii* and *N. caninum*, in wild ruminants worldwide and in Europe is estimated at 23–63.5% and 0.88–23.88%, respectively (20–22). Older and female animals have occasionally been associated with an increased susceptibility to infection with *T. gondii* (23–26). A Slovenian study on *T. gondii* in wild boars (*Sus scrofa*) showed a 62% seroprevalence and a significant risk of infection with increasing age and weight (27). In addition, a seroprevalence of *T. gondii* and *N. caninum* in some wild ruminant species was also reported in a Slovenian zoo, which was 13–60% and 0–13%, respectively (28).

*Brucella* spp., *Coxiella burnetii* and *Chlamydophila abortus* are among the significant pathogens causing abortions in ruminants. Currently, *Brucella* spp. are rarely found in most EU Member States. Nevertheless, these species possess a significant zoonotic risk, particularly *B. abortus/melitensis*, and continue to be detected in ruminants originating from Greece, Italy, Spain, Portugal, and other Mediterranean areas beyond the borders of the European Union (29). Infections with *B. abortus* and *B. suis* have been documented in various wildlife species, while *B. melitensis* has only rarely been detected in wildlife. In Europe, only isolated cases of infection have been reported, particularly in chamois and Alpine ibex (30–32). In contrast to *Brucella* spp. most outbreaks of Q fever in humans caused by *C. burnetii* are associated with domestic and wild ruminants, with seroprevalence ranging from 0 to 29% in wild ruminants (33–35) and up to 75% in domestic ruminants (34, 36–38). This may suggest that wild ruminants could also contribute to the maintenance of *C. burnetii* in nature (37). *Chlamydophila abortus*, also a common cause of abortion in sheep and goats (38), has been detected in 0–40% of wild ruminants. Although it is evident that wildlife can serve as reservoir for Chlamydiaceae, the potential significance for human and animal health remains uncertain (39, 40).

*Mycobacterium avium* subsp. *paratuberculosis* (MAP) and *Mycobacterium bovis* are additional pathogens that have a significant impact on the economic sustainability of livestock farming by reducing production levels and fertility rates (41, 42). Slovenia is officially free of bovine tuberculosis (43). Most member states of the European Union have also achieved this status. In our neighboring country Italy, on the other hand, there are still outbreaks of bovine tuberculosis in some provinces (44). Tuberculosis in wildlife can affect humans and a wide range of domestic and wild mammals and is also a significant problem for global conservation efforts. Numerous wildlife species play a role in the persistence and spread of tuberculosis. It is therefore important to systematically monitor both wild and domestic hosts in order to effectively implement prevention and control strategies (45, 46). In a study carried out in Slovenia on 306 apparently healthy wild animals of 13 different species, mycobacteria were isolated in 36 (11.8%) animals of 5 species, namely red deer, roe deer, fallow deer, wild boar and golden jackal (47). In contrast to the official status of bovine tuberculosis, the seroprevalence of MAP in Slovenian dairy farms was reported to be 18.49% at herd level and 3.96% at animal level according to a study by Kusar et al. (48). In addition, the molecular prevalence of MAP is higher on larger farms, as Logar et al. (49) found.

Currently, the number of medium-sized family farms in Slovenia is decreasing, which has an impact on environmental sustainability. It is possible that some of the remaining farms will switch from exclusively keeping animals indoors to more animal-friendly free-range farming with more access to pasture. As wildlife often share pastures with livestock, pathogens can be transmitted in both directions. It is therefore important to continuously monitor both populations to ensure good health and reproductive fitness of the herds as well as safe venison for human consumption.

The aim of the present study was to: 1. Determine seroprevalences of *T. gondii* and other important pathogens in wild ruminants; 2. Assess species, age, sex, and coinfection as possible risk factors for infection; 3. Evaluate wild ruminants as a potential reservoir for diseases of medical and veterinary importance.

## 2 Materials and methods

### 2.1 Materials

**Figure 1:**
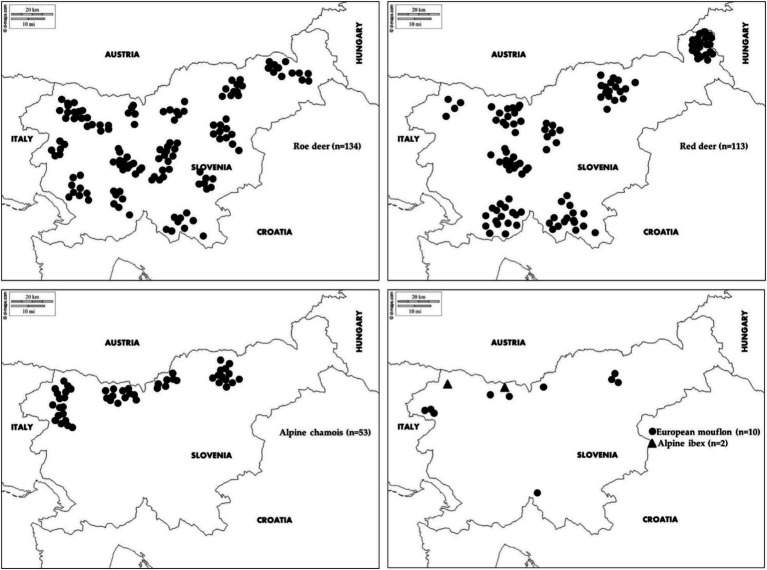
Geographical locations of samples collected from roe deer, red deer, Alpine chamois, mouflon and Alpine ibex in Slovenia.

Wild ruminant serum samples (*n* = 312) were collected during the 2017 and 2018 hunting season from apparently healthy free-ranging animals throughout Slovenia (Figure 1). Game wardens and hunters were asked to submit samples from animals shot during the regular annual cull. In Slovenia, the regular annual culling months are from May to December (roe deer), from July to January (red deer) and from August to December (Alpine ibex, chamois and mouflon). The blood was collected shortly after death from the jugular vein or the heart by trained hunters equipped with field sampling kits. Due to autolysis and post-mortem changes, the samples were checked at the Veterinary faculty and haemolysed samples were rejected at the pre-analysis stage. Samples were collected from 134 roe deer, 113 red deer, 53 Alpine chamois, 10 European mouflons and 2 Alpine ibex of various sex and age.

The samples were transported to the laboratory, where they were centrifuged for 15 min at 4000 rpm. Sera were transferred with serum pipettes into sterile Eppendorf tubes and stored at −20°C until testing. Only animals whose species, sex, and age were recorded by the hunters were used for this study. The age of the animals was estimated after the time of culling by an authorized committee of hunters during the mandatory annual inspection of harvested ungulates. For roe deer, eruption patterns and tooth wear were used to estimate age, while for chamois and mouflon the horn growth ring method was used. Depending on the age of the animals, they were categorized into one of two age groups: young (<1 year old) and adult (>1 year old). Most samples (*n* = 254) were obtained from animals older than 1 year, namely from 1 to 18 years, with an average age of 2 years.

### 2.2 Methods

#### 2.2.1 Serological methods

Commercial test kits were used for the detection of antibodies to the selected pathogens. Antibodies against *T. gondii* were tested using ID Screen® Toxoplasmosis Indirect Multi-species ELISA (ID.vet, Montpellier, France), which detects antibodies against *T. gondii* P30 antigen in sera, plasma, and meat juices. Antibodies to *N. caninum* were tested using ID Screen® *Neospora caninum* Competition kit (ID.vet, Montpellier, France). Sera for brucellosis were screened by rose bengal plate test (RBPT) using Pourquier® Rose Bengal Anti-gen (IDEXX, Montpellier, France). It was followed by indirect-Enzyme Linked Immuno-sorbent Assay (iELISA) using ID Screen® Brucellosis Serum Indirect Multi-species (ID.vet, Montpellier, France). Antibodies against *C. abortus* were screened using ID Screen® *Chlamydophila abortus* Indirect Multi-species (ID.vet, Montpellier, France). It is used to detect anti-*C. abortus* IgG antibodies against a synthetic antigen from a major outer-membrane protein (MOMP), which is specific to *C. abortus* in serum or plasma. Antibodies against *C. burnetii* were tested using Q-Fever (*Coxiella burnetii*) Antibody Test Kit (IDEXX, Liebefeld-Bern, Switzerland). For the detection of specific antibodies against MAP we used IDEXX Paratuberculosis screening Ab test (IDEXX Laboratories, Inc., Westbrook, ME, USA). Antibodies to *Mycobacterium tuberculosis* complex were screened in MPB83 antigen sandwich ELISA using INGEZIM Tuberculosis DR ELISA kit (Ingenasa, Spain). All tests are suitable for the detection of antibodies in ruminant serum and assays were performed according to the manufacturer’s instructions. Data about sensitivity and specificity for each commercial kit should be available on the manufacturer’s website. Thresholds were determined for each kit following the manufacturer’s instructions. Doubtful results were considered negative.

#### 2.2.2 Statistical analyses

Data were summarized as frequencies (%) with corresponding (exact) binomial 95% confidence intervals.

Differences between the groups (positive vs. negative) for gender (male, female), age (young, adult), gender vs. age (adult female, adult male, young female, young male), and species (roe deer, red deer, alpine chamois, mouflon, alpine ibex) were tested using Fisher’s exact test; for larger than 2 by 2 tables we used the approach proposed by Mehta and Patel (50).

The association between the outcome and a predetermined set of covariates was estimated using multiple binomial penalized regression utilizing Firth’s penalty (51). Results are presented as odds ratios (OR) with 95% confidence intervals (CI). CIs were obtained by profiling the (penalized) likelihood. A *p*-value lower than 0.05 was considered statistically significant. R statistical language (version 3.6.1) was used for the analyses. R package logistf was used to fit the models (52).

## 3 Results

The results for all pathogens tested in 312 wild ruminants are shown in Table 1. *Toxoplasma gondii* proved to be the most prevalent pathogen with 108/312 (34.62%) seropositive animals. Females showed a higher prevalence than males (73/182 vs. 35/130, *p* = 0.02). In relation to age and sex (*p* = 0.02), adult and young females had a prevalence of 40.91% (63/154) and 40% (12/30), respectively. Young males had a similar, but lower prevalence of 35.71% (10/28), while adult males had the lowest prevalence of 23% (23/100). The most affected species (*p* < 0.01) was roe deer with 46.27% (62/134), followed by European mouflon and red deer with 30% (3/10) and 28.32% (32/113), respectively. Alpine chamois had a *T. gondii* seroprevalence of 20% (11/53), while both Alpine ibexes were seronegative (0/2). *Coxiella burnetii* antibodies were detected in 7 samples (6 females, 1 male). It was detected in one Alpine ibex (50%, 1/2), one European mouflon (10%, 1/10), in two Alpine chamois (3.77%, 2/53), three roe deer (2.24%, 3/134) and in none of the sampled red deer (0/113). *Neospora caninum* and *C. abortus* antibodies were detected in three samples each from roe deer and red deer, while only red deer (1.77%, 2/113) were seropositive for *Mycobacterium bovis*. Antibodies against *Brucella* spp. and MAP were not detected in any of the samples.

**Table 1:** Univariate analysis of various diseases in the Slovenian wild ruminant population detected by serology.

	*Toxoplasma gondii*	*Neospora caninum*	*Coxiella burnetii*	*Brucella* sp.	*Chlamydophila abortus*	MAP	*Mycobacterium tuberculosis* complex
All (*n* = 312)	108 (34.62%, CI 29.34–40.19)	3 (0.96%, CI 0.19–2.79)	7 (2.24%, CI 0.9–4.57)	0 (0%, CI 0–1.18)	3 (0.96%, CI 0.19–2.79)	0 (0%, CI 0–1.18)	2 (0.64%, CI 0.07–2.3)
Gender	*p* = 0.02	*p* = 0.57	*p* = 0.25	/	*p* = 1	/	*p* = 1
Female (*n* = 182)	73 (40.11%, CI 32.92–47.62)	1 (0.55%, CI 0.01–3.03)	6 (3.3%, CI 1.21–7.04)	/	2 (1.1%, CI 0.13–3.92)	/	1 (0.55%, CI 0.01–3.03)
Male (*n* = 130)	35 (26.92%, CI 19.52–35.41)	2 (1.54%, CI 0.18–5.45)	1 (0.77, CI 0.01–4.22)	/	1 (0.77%, CI 0.01–4.21)	/	1 (0.77%, CI 0.01–4.22)
Age	*p* = 0.55	*p* = 0.46	*p* = 1	/	*p* = 0.46	/	*p* = 1
Young (*n* = 58)	22 (37.93%, CI 25.51–51.63)	1 (1.72, CI 0.04–9.24)	1 (1.72%, CI 0.04–9.24)	/	1 (1.72%, CI 0.04–9.24)	/	0 (0%, CI 0–6.17)
Adult (*n* = 254)	86 (33.85%, 28.06–40.04)	2 (0.79, CI 0.09–2.82)	6 (2.36%, CI 0.87–5.07)	/	2 (0.79%, CI 0.09–2.82)	/	2 (0.79%, CI 0.09–2.82)
Species	*p* = 0.00	*p* = 1	*p* = 0.00	/	*p* = 0.8	/	*p* = 0.36
Roe deer (*n* = 134)	62 (46.27%, CI 37.62–55.08)	2 (1.49%, CI 0.18–5.29)	3 (2.24%, CI 0.46–6.41)	/	1 (0.75%, CI 0.01–4.08)	/	0 (0%, CI 0–2.72)
Red deer (*n* = 113)	32 (28.32%, CI 20.24–37.57)	1 (0.88, CI 0.02–4.84)	0 (0%, 0–3.22)	/	2 (1.77%, CI 0.21–6.25)	/	2 (1.77%, CI 0.21–6.25)
Alpine chamois (*n* = 53)	11 (20.75%, CI 10.84–34.11)	0 (0%, CI 0–6.73)	2 (3.77%, CI 0.46–12.98)	/	0 (0%, CI 0–6.73)	/	0 (0%, CI 0–6.73)
Mouflon (*n* = 10)	3 (30%, CI 6.67–65.25)	0 (0%, CI 0–30.85)	1 (10%, CI 0.25–44.51)	/	0 (0%, CI 0–30.85)	/	0 (0%, CI 0–30.85)
Alpine ibex (*n* = 2)	0 (0%, CI 0–84.19)	0 (0%, CI 0–84.19)	1 (50%, CI 1.25–98.75)	/	0 (0%, CI 0–84.19)	/	0 (0%, CI 0–84.19)
Sex:age	*p* = 0.02	*p* = 0.49	*p* = 0.51	/	p = 0.4	/	*p* = 1
F: AD (*n* = 154)	63 (40.91%, CI 33.06–49.11)	1 (0.65%, CI 0.01–3.57)	5 (3.25%, CI 1.06–7.42)	/	1 (0.65%, CI 0.01–3.57)	/	1 (0.65%, CI 0.01–3.57)
M: AD (*n* = 100)	23 (23%, 15.17–32.49)	1 (1%, CI 0.02–5.45)	1 (1%, CI 0.02–5.45)	/	1 (1%, CI 0.02–5.45)	/	1 (1%, CI 0.02–5.45)
F: Y (*n* = 30)	12 (40%, 22.65–59.4)	1 (3.33%, CI 0.08–17.22)	0 (0%, CI 0–11.58)	/	0 (0%, CI 0–11.57)	/	0 (0%, CI 0–11.58)
M:Y (*n* = 28)	10 (35.71%, 18.64–55.94)	0 (0%, CI 0–12.35)	1 (3.57%, CI 0.09–18.35)	/	1 (3.57%, CI 0.09–18.35)	/	0 (0%, CI 0–12.35)

*p-*values (obtained by using Fisher’s exact test) are not adjusted for multiple comparisons and should be interpreted for exploratory purposes only.

As most pathogens had a low prevalence, a multivariate analysis was only performed for *T. gondii* (Table 2). The results show that female animals have a significantly higher risk of *T. gondii* infection (*p* = 0.03), with an odds ratio of 1.77. Adult animals are also more frequently infected, but the effect was not significant (*p* = 0.20). Among the animal species, red deer and chamois are both less susceptible to infection than roe deer (*p* < 0.01). No statistical significance was observed between the infection rate of roe deer versus European mouflon (*p* = 0.28) and Alpine ibex (*p* = 0.32).

The prevalence of *T. gondii* in 3 most prevalent species based on animals age and sex showed females animals to be more likely to be positive for *T. gondii* in roe deer (*p* = 0.01) and alpine chamois (*p* = 0.15), although the later was not statistically significant (Table 3). Older animals were more likely (not statistically significant) to be seropositive for *T. gondii* than younger animals in red deer (*p* = 0.25) and alpine chamois (*p* = 0.2), compared to roe deer (*p* = 0.97) (Table 3).

**Table 2:** Risk factors for *Toxoplasma gondii* seroprevalence by sex, age, species and comparison between species of wild ruminants.

Wild ruminants^a^^b^	Estimate (SE)	Odd ratio	Confidence interval (95%)	*p*-value
Gender (female vs. male)	0.57 (0.26)	1.77	1.07–2.96	0.03
Age (young vs. adult)	−0.40 (0.31)	0.67	0.36–1.25	0.20
Species				0.01
Red deer vs. Roe deer	−0.85 (0.28)	0.43	0.24–0.73	<0.01
Alpine chamois vs. Roe deer	−1.06 (0.38)	0.35	0.15–0.72	<0.010
European mouflon vs. Roe deer	−0.71 (0.68)	0.49	0.11–1.76	0.28
Alpine ibex vs. Roe deer	−1.38 (1.56)	0.25	0.00–3.27	0.32

^a^McFadden’s pseudo R-squared = 0.05; the pseudo R^2^was defined as 1-log(L_1_)/log(L_0_), where L_1_ and L_0_ are the likelihoods of the full model and the intercept-only model, respectively.

^b^p-value for the likelihood ratio test comparing the full model and intercept only model < 0.01.

**Table 3:** Risk factors for *Toxoplasma gondii* seroprevalence by sex and age by different species.

**Wild ruminants**	**Estimate (SE)**	**Odd ratio**	**Confidence interval (95%)**	***p*-value**
Roe Deer (*n* = 134)				
Gender (female vs. male)	0.89 (0.37)	2.43	1.19–5.10	0.01
Age (young vs. adult)	0.02 (0.53)	1.02	0.36–2.97	0.97
Red Deer (*n* = 113)				
Gender (female vs. male)	−0.22 (0.42)	0.80	0.35–1.87	0.61
Age (young vs. adult)	−0.51 (0.45)	0.60	0.24–1.46	0.25
Alpine chamois (*n* = 53)				
Gender (female vs. male)	0.97 (0.68)	2.65	0.70–10.83	0.15
Age (young vs. adult)	−1.03 (0.80)	0.35	0.07–1.81	0.20

The subgroup analyses were performed for the 3 most commonly represented species in our study. The p-values are not adjusted for multiple comparisons and should be interpreted for explorative purpose only.

## 4 Discussion

Wildlife diseases manifest in different forms and affect a wide range of animal species worldwide. Diseases surveillance is therefore an important tool that provides crucial information on the health status of animal populations and thus ensures the protection of human health. The use of postmortem examinations of wildlife carcasses found in nature or carcasses of animals harvested due to observed poor health offers valuable insight into the general health of a population and the influence of sex, age, and geographical distribution on the cause of death (1, 53). The present study investigated the seroprevalence of seven zoonotic and/or economically important pathogens of apparently healthy animals from five different species of wild ruminants that may have profound effect on human/livestock health and economic viability of the farms. This is the first study investigating the seroprevalence and related risk factors of *T. gondii*, *N. caninum, C. burnetii, Brucella* spp., *C. abortus*, MAP and *M. bovis* in wild ruminants in Slovenia.

Game meat is a precious source of protein that is increasing in popularity (54). Due to wild ruminant expansion, especially roe deer, the availability of game meat is high (5, 6, 54). However, an apparently healthy animal can harbour many diseases (54) that pose an immediate danger to livestock, hunters or those who handle the carcasses, such as toxoplasmosis, brucellosis, Q fever, chlamydiosis and tuberculosis (27, 55–57). They can also harbour pathogens that can indirectly affect the health of livestock (54), such as neosporosis and paratuberculosis (21, 58). The seroprevalence of many of these diseases is usually much higher in domestic ruminants, than in wild ruminants (59, 60). Ruminants generally exhibit a higher susceptibility to MAP infections compared to other animal species (61). In Slovenian cattle, the reported prevalence of paratuberculosis is about 20% (48), whereas it is 0% in the wild ruminants observed in this study. It appears that a low seroprevalence of MAP in wild ruminants is not uncommon in Europe. Tavernier et al. (10) reported a prevalence of 4.1% in the Belgian roe deer population, while a recent Italian study found a similarly low overall seroprevalence of 5.9% in red deer using different diagnostic tools (62). In addition, Machackova et al. (63) found no significant difference in the seroprevalence reported for MAP (5.3%) between farmed and wild red deer.

In this study we reported a 2.24% seroprevalence against *C. burnetii* in wild ruminants, which is considerably lower comparing to a Slovenian study in sheep with a prevalence of up to 40.4% (37). However, it should be acknowledged that sheep from the study of Knap et al. (37) most likely represent a much higher seroprevalence than what would be a true seroprevalence in Slovenia as only Q fever positive herds with a history of reproductive failure were tested. Another possible reason for the low prevalence in wild ruminants could be that some of the infected animals do not seroconvert (64). Ticks taken from red deer and wild rabbit in Spain were shown to transmit the disease during blood feeding (65) and a study from Portugal reported a seroprevalence of 1.9% in red deer (66). The reported prevalence is low, but still higher than reported in our study, where all samples off red deer were negative for Q fever antibodies. Following Q fever, chlamydiosis is often considered the second most common abortive pathogen in small domestic ruminants, with prevalence rates ranging from 18.2 to 96.5% (38, 67, 68). Only limited information is available on chlamydiosis in wild ruminants. Serological techniques to identify the specific chlamydial species are lacking, although serological studies conducted in Europe indicate that wild ruminants are a possible but less likely reservoir for chlamydial infections (69, 70). In our study, the presence of *C. abortus* antibodies was confirmed in only one sample from red deer and two samples from roe deer, which corresponds to 0.96% of the tested wild ruminants. The low prevalence of *C.abortus* in wild ruminants is therefore similar to the Italian study, in which no seropositive samples were found in red deer (9), while in Belgium Tavernier et al. (10) reported a prevalence of *C. abortus* of 6.7% in roe deer.

The infective potential of *N. caninum* extends to various hosts, yet its most pronounced effects are evident in cattle and dogs. There was no evidence for the occurrence of vertical *N. caninum* infections and their significance in wildlife, and the results of Zanet et al. (71) indicated the possibility of congenital transmission of *N. caninum* in roe deer, wild boar and red fox. Dubey et al. (72) reported the clinical case of neosporosis in deer, but *N. caninum* antibodies and/or DNA have been found in several European wild ruminant species, i.e., red deer (7, 8, 73), roe deer (7, 73), fallow deer (74), Alpine chamois (69, 73, 75), European mouflon (76), European bison (*Bison bonasus bonasus*) (77), Alpine ibex (75) and Spanish ibex (*Capra pyrenaica hispanica*) (78). The seroprevalence of *N. caninum* in wild ruminants in Slovenia is 0.96%. The presence of antibodies was confirmed in both autochthonous deer species, roe deer (1.4%) and red deer (0.8%). Currently, no published data are available on livestock in Slovenia. This finding emphasizes the need for further research to establish the role of *N. caninum*, as it is widely believed that intensive livestock farming creates the conditions for the occurrence and spread of this unicellular parasite (59).

The only pathogen that is hardy affected by human land management is *T. gondii*. It is possibly the most common parasite within the domestic and sylvatic cycle (79). Domestic and wild ruminants can serve as a source of infection for animals and humans through the consumption of meat. The primary route of infection for herbivores is the ingestion of oocysts, which are shed into the environment by their definitive hosts, which in Central Europe are the domestic cat (*Felis catus*), the wild cat (*Felis silvestris*) and the Eurasian lynx (*Lynx lynx*) (11, 12, 14, 80). *Toxoplasma gondii* is also the pathogen with the highest prevalence in wild ruminants in our study. The seroprevalence in our study was 46.2% in roe deer and 28.3% in red deer, which is above the European average for both species of 29 and 15%, respectively (11). A high seroprevalence of *T. gondii* was also found in the Slovenian wild boar population at 62% (27). An overall high seroprevalence in Slovenian wild animal population would need further research as it is surprising. Slovenian climate, which is Mediterranean/Continental/pre-Alpine might allow for *T. gondii* to thrive (27). Risk factors associated with higher *T. gondii* exposure are usually species, sex, age, and geographical distribution (23, 26, 81–83). Roe deer are more likely to be *T. gondii* positive than red deer and chamois. These results confirm the findings of researchers in Denmark and Norway, where roe deer were more likely to be *T. gondii* positive than fallow deer (83), moose, red deer or reindeer (23). The most consistent risk factor associated with increased *T. gondii* seroprevalence is usually the increasing age of the animals (23, 24, 84–88) as antibodies can persist for several years and the likelihood of seroconversion increases over an animal’s lifetime (80). However, our study did not show any significance due to the age of the animal when all species were included in the analysis. Further analysis of the most representative species (roe deer, red deer, alpine chamois) in our study, showed that younger animals were less likely (although not reaching statistical significance) to be positive than older animals for red deer and alpine chamois, while for roe deer the age seemed to be unimportant. On the other hand, the sex of the animal is not consistently associated with a higher prevalence of *T. gondii*. Several studies found no correlation (23, 84, 86, 88), including our study in wild boar population (27). Only a few studies found that females are more frequently infected than males (24, 26, 89), while one study found that a male population of white-tailed deer was significantly more seropositive than females (24). In our study, females were significantly more seropositive for *T. gondii* than males (*p* = 0.03). When analysing the relationship between age and gender, it was found that adult and young females had an almost identical prevalence rate (40.91% vs. 40%), young males had a similar but lower prevalence of 35.71%. The difference was significant in adult males, who had a prevalence of only 23% (*p* = 0.02). The lower prevalence in adult males does not fit with the theory of increased exposure over time theory. Nor does it fit with the same seroprevalence in young and adult females. It could be, that infection with *T. gondii* has no effect on the survival of females, but it does on the survival of males. A more detailed species specific analysis in our study revealed a similar effect of gender for roe deer and alpine chamois (more positive between females than males), but no association was observed for red deer. *Toxoplasma gondii* has been found to affect the sex ratio in humans and mice, with more males being born than females (90, 91). Studies in mice showed that males infected with *T. gondii* had decreased levels of hormones and other parameters that impaired the animal’s general reproductive capacity (92). Several studies have also confirmed that wildlife generally found at lower altitudes and below the tree line such as roe deer and red deer, have a high seroprevalence of *T. gondii*. A lower prevalence was found in species that favour rocky terrain at moderately high altitudes such as chamois (11%) and mouflon (3%) as well as alpine ibex (0%). This finding confirms the significant effect of altitude as a protective factor due probably to less contact with oocysts in areas of high altitude, as animals from higher altitudes were less likely to be seropositive (13, 80, 93). Scherrer et al. (80) reported a high seroprevalence of 82% in Eurasian lynx, one of the potential definitive hosts of *T. gondii* in Europe. This may indicate a higher prevalence in roe deer and chamois, the main pray animals of lynx. In this study, we were unable to evaluate coinfection as a risk factor for *T. gondii* infection, as the prevalence of other pathogens was low in the animals examined. This indicates that apart from *T. gondii*, all other investigated pathogens are not of major importance in the sylvatic cycle in Slovenia and thus do not represent an important source of disease for humans or animals. Regardless, precautions with raw and undercooked meat, including game meat, are still needed when handling wildlife and their carcasses.

## 5 Conclusion

In conclusion, the health status of wild ruminants remains largely unknown, but studies, including ours, suggest that some pathogens are less prevalent than in domestic ruminants. Surveillance of infective agents in wildlife provides insight into the current epidemiological situation of selected pathogens. It can help prevent spillovers from wildlife to humans and livestock and detect spillbacks from humans and livestock to wildlife. *Toxoplasma gondii* was the most predominant pathogen investigated with a significantly higher seroprevalence in roe deer and females. The lower prevalence in adult males provides an interesting research window for future studies. The results of our study also emphasize the potential risk for hunters, veterinary pathologist, farmers and general public associated with the consumption of venison and handling of carcasses from wild ruminants. Personal protective measures should be taken and proper food preparation are suggested as a preventive measure, no less important than when handling raw meat from domestic animals.

## Data availability statement

The raw data supporting the conclusions of this article will be made available by the authors, without undue reservation available upon request.

## Ethics statement

Ethical approval was not required for the study involving animals in accordance with the local legislation and institutional requirements because the approval of the Ethics Committee/Welfare Authority was not required as all samples were taken post-mortem.

## Author contributions

DŽV: Conceptualization, Data curation, Investigation, Visualization, Writing – original draft, Writing – review & editing. BK: Conceptualization, Data curation, Formal analysis, Investigation, Methodology, Validation, Writing – review & editing. RB: Data curation, Formal analysis, Methodology, Software, Writing – review & editing. GV: Conceptualization, Data curation, Funding acquisition, Investigation, Project administration, Resources, Supervision, Visualization, Writing – review & editing. PB: Conceptualization, Data curation, Funding acquisition, Visualization, Writing – original draft, Writing – review & editing.
